# Intake of allergenic foods at 1.5 years and 3 years of age in a general child population in Japan: a cross-sectional study

**DOI:** 10.1265/ehpm.22-00213

**Published:** 2023-01-21

**Authors:** Takafumi Takase, Mizuho Nagao, Rei Kanai, Takahiro Nishida, Tomoyuki Arima, Fumiko Iwai, Shingo Yamada, Makiko Nakamoto, Masahiro Hirayama, Takao Fujisawa

**Affiliations:** 1 Allergy Center and Department of Clinical Research, National Hospital Organization Mie National Hospital; 2 Department of Pediatrics, Mie University Graduate School of Medicine

**Keywords:** Atopic dermatitis, Asthma, Epidemiology, Food hypersensitivity, Surveys, Questionnaires

## Abstract

**Background:**

Recent studies indicate that the timing of introduction of potentially allergenic food is crucial for the development of food allergy in children. This cross-sectional study aimed to clarify the reality of allergen food intake in a general population of young children in Japan.

**Methods:**

A questionnaire survey of caregivers was conducted at health checkups for 1.5-year (18-month)-old and 3-year-old children in the fall of 2020. The caregivers were asked about (1) the presence/absence of allergic disease symptoms based on the ISAAC questionnaire, and (2) foods that caregivers avoided giving their children. Ordinal logistic regression analyses were periformed to determine factors associated with food avoidance.

**Results:**

Questionnaires were distributed to 1720 caregivers, and 1603 (93%) responded. The responders consisted of 771 and 832 caregivers who participated in 1.5-year-old and 3-year-old checkups, respectively. The prevalence of allergic diseases was comparable to recent epidemiological studies in Japan, indicating that the population may be representative. At 1.5 years old, more than 50% of the children were not exposed to peanuts, tree nuts, fish eggs, shellfish, and buckwheat. At 3 years old, the avoidance rates of the foods had decreased but were still between 18.8% and 32.0%. On the other hand, the avoidance rates of chicken egg and cow’s milk, the top 2 common allergenic foods in Japan, were much lower at 2.8% and 1.5% at 1.5 years, and they decreased to 1.4% and 0.7% at 3 years old, respectively. Ordinal logistic analysis showed that avoidance of chicken egg, cow’s milk, and wheat was associated with food allergy diagnosis and chicken egg avoidance with eczema, but avoidance of other foods showed no associations with any risk factors for food allergy.

**Conclusion:**

Caregivers avoided giving various foods, independent of allergy risk factors, to their young children. Since delayed introduction of an allergenic food has been reported to increase the risk of developing an allergy to the food, the results warrant future investigation of the development of food allergies in relation to current eating habits and recommendations.

**Supplementary information:**

The online version contains supplementary material available at https://doi.org/10.1265/ehpm.22-00213.

## Background

Recently, the prevalence of food allergies has increased rapidly in developed countries [1–3]. Unlike for asthma and other allergic diseases, there is no pharmacotherapy to control food allergies, and dietary restriction avoiding the causative food is a significant burden on patients [4]. Accordingly, prevention of food allergy has been the main focus.

In this context, delaying the introduction of allergenic foods for high-risk infants—for example, refraining from feeding dairy products until 1 year, chicken eggs until 2 years, and peanuts, tree nuts, and fish until 3 years of age—was recommended by the American Academy of Pediatrics in 2000 [5] and other Pediatric organizations, including in Japan. However, recent epidemiological studies have demonstrated positive associations between early—not delayed—introduction of allergenic foods and a low prevalence of food allergy [6, 7]. Then, in 2015, the LEAP Study [8] in England proved that early consumption of peanuts by high-risk infants with eczema prevented peanut allergy. Similarly, in 2017, the PETIT Study in Japan [9] demonstrated that early consumption of chicken egg prevented egg allergy. These results completely contraindicated the old recommendations of delayed introduction, leading to recommendation of early introduction of peanuts for high-risk infants in the US [3] and for the general population in Australia [10]. In Japan, based on the above evidence, the “Breastfeeding and Weaning Support Guide” [11] states that “there is no scientific evidence to support the prevention of food allergy by delaying weaning or introduction of certain foods out of concern for food allergy,” and it recommends that allergenic chicken egg be started at around 5 to 6 months of age. The Japanese Guidelines for Food Allergy [12] also support that recommendation and further recommends that high-risk infants with atopic dermatitis be introduced to small amounts of chicken egg from 6 months of age after adequate control of atopic dermatitis under medical supervision.

Subsequent to these changes in recommendations, the dietary habits in Australia have drastically changed, and the rate of introduction of peanuts in the first year of life increased from 28% in 2007 to 2011 to 89% in 2018 to 2019 [13]. In the US the timing of introduction differs widely among the races [14]. In Japan, the actual status of allergenic food intake in infants and toddlers has not been studied. Although multiple factors such as early eczema [15], the environment [16], microbiome [17], and genes [18] are involved in the development of food allergy, the importance of the timing of initial allergen intake is undisputed in light of those previous studies.

Thus, we investigated the status of intake of various allergenic foods in a general population of Japanese toddlers in relation to the prevalence of childhood allergic disease.

## Methods

This cross-sectional study used data from an administrative survey by the Health Promotion Division, Department of Medical Health, Mie Prefectural Government, in collaboration with Allergy Center, National Hospital Organization Mie National Hospital. The survey was conducted in accordance with the Basic Law on Measures Against Allergic Diseases [19].

### Surveyed subjects

Caregivers of children who underwent health checkups at 1.5 and 3 years of age in Tsu City and Suzuka City, Mie Prefecture, from September through November 2020 were asked to fill out a self-administered questionnaire.

### Questionnaire

The questionnaire consisted of questions about the age of the child (1.5 or 3 years); gender; modified ISAAC (International Study of Asthma and Allergies in Childhood) questions [20] regarding wheezing, eczema, rhinitis and rhinoconjunctivitis; a question regarding the presence or absence of physician-diagnosed food allergy; feeding/avoidance of various allergenic foods; and administrative questions pertaining to the medical needs regarding allergic diseases and the Corona virus disease-19 (COVID-19) pandemic (Table E1).

The modified ISAAC questions regarding wheezing, eczema and rhinitis asked about the presence or absence of corresponding symptoms “at any time in the past”, i.e., “wheezing ever”, “eczema ever”, and “rhinitis ever”. The question regarding symptoms “in the last 12 months” was omitted because of considerable redundancy with “at any time in the past”. For rhinoconjunctivitis, on the other hand, the caregivers were asked about symptoms “in the last 12 months” time-frame for specificity, because rhinitis symptoms such as runny nose and sneezing at a young age are hard to distinguish from common colds.

Regarding food intake, the responding caregivers were asked to indicate their basic approach: not eating at all because of food allergy concerns (or diagnosis), eating moderately, or eating normally. Allergenic foods were categorized as 1) foods reported as common allergens in Japan [12], such as chicken eggs, cow’s milk, wheat, tree nuts, peanuts, fruits, fish eggs (salmon roe), crustaceans, buckwheat, soybeans, and fish; and 2) less common allergenic foods, such as beef, pork, chicken, vegetables, and sesame seeds, which cover all kinds of foods that Japanese toddlers normally eat. Since blue-flesh fish are commonly—without any scientific basis—believed to cause more allergies than white-flesh fish in Japan, those 2 kinds of fish were listed separately.

### Statistical analysis

Descriptive statistics were used to summarize the number and percentage of responses to each question. Binary logistic regression was used to analyze for association of questionnaire-identified allergic diseases with known risk factors. Ordinal logistic regression models for food avoidance were used and odds ratios (ORs) and 95% confidence intervals (CI) were estimated. Outcomes (avoidance of each food at 3 levels, “eat with no restriction”, “eat modestly”, and “never eat”) and explanatory variables (known risk factors of food allergy and sources of medical information that caregiver seek) were analyzed in separate models. JMP^®^ 16.20 was used for the analyses.

### Ethics

This study was conducted as an administrative survey of the Mie Prefecture Allergic Disease Control Project and was approved by the Ethics Committee of the National Hospital Organization Mie National Hospital (approval number 202201). All respondents gave written informed consent. No personal information was collected.

## Results

### Background of the children and prevalence of allergic diseases

The survey forms were distributed to 1720 caregivers of Japanese toddlers who received health checkups at 1.5 years and 3 years of age, and 1603 (93.2%) responded. The characteristics of the children are summarized in Table 1. The gender ratio was almost 1:1. The overall prevalence of wheezing was 20.2%, with larger proportions in boys and 3-year-olds. Rhinitis symptoms were reported for 40.4%, of which 6.9% had current rhinoconjunctivitis. “Eczema ever” was reported for 14.6% of the subjects, and a food allergy diagnosis was reported for 10.8%.

**Table 1:** Characteristics of the children: prevalence of allergic diseases

	**1.5-year-old checkup**	**Not stated**	**3-year-old checkup**	**Not stated**	**Total, n (%)**	**Not stated**
N	771	-	832	-	1603	-
Gender, boys/girls (n)	319/355	97	383/377	72	702/732	169

**Wheezing ever,** %	**16.1%**	5	**24.1%**	3	**20.2%**	8
boys/girls, %	20.6%/12.7%	3/2	25.2%/23.1%	2/1	23.1%/18.1%	5/3
not stated, %	13.4%	0	22.2%	0	17.2%	0

**Rhinitis ever** (%)	**38.4%**	1	**42.2%**	1	**40.4%**	2
boys/girls, %	39.9%/35.5%	1/0	42.8%/41.2%	0/0	41.5%/38.4%	1/1
not stated, %	44.3%	0	44.4%	0	44.3%	0

**Current rhinoconjunctivitis** (%)	**4.1%**	16	**9.5%**	18	**6.9%**	34
boys/girls, %	5.8%/2.9%	7/8	12.8%/6.2%	9/7	9.6%/4.6%	16/15
not stated, %	3.1%	1	8.6%	2	5.4%	3

**Eczema ever** (%)	**15.0%**	3	**14.2%**	6	**14.6%**	9
boys/girl, %	17.3%//14.4%	1/2	15.3%//12.8%	3/3	16.2%/13.6%	4/5
not stated, %	9.3%	0	15.3%	0	11.8%	0

**Food allergy diagnosis,** n (%)	**10.6%**	4	**11.1%**	3	**10.8%**	7
boys/girls, %	12.2%/9.9%	0/3	11.0%/11.2%	1/1	11.6%/10.6%	1/4
not stated, %	7.3%	1	11.3%	1	9.0%	2

### Associations of allergic diseases with possible risk factors

Logistic regression analysis was performed to examine for associations of questionnaire-identified allergic diseases with known risk factors (Table E2). Food allergy diagnosis was significantly associated with eczema and wheezing, while wheezing was associated with eczema, food allergy, and rhinitis. Eczema was associated with wheezing, food allergy, and rhinitis. Rhinitis was associated with eczema and wheezing (Table E2).

### Food avoidance

**Fig. 1:**
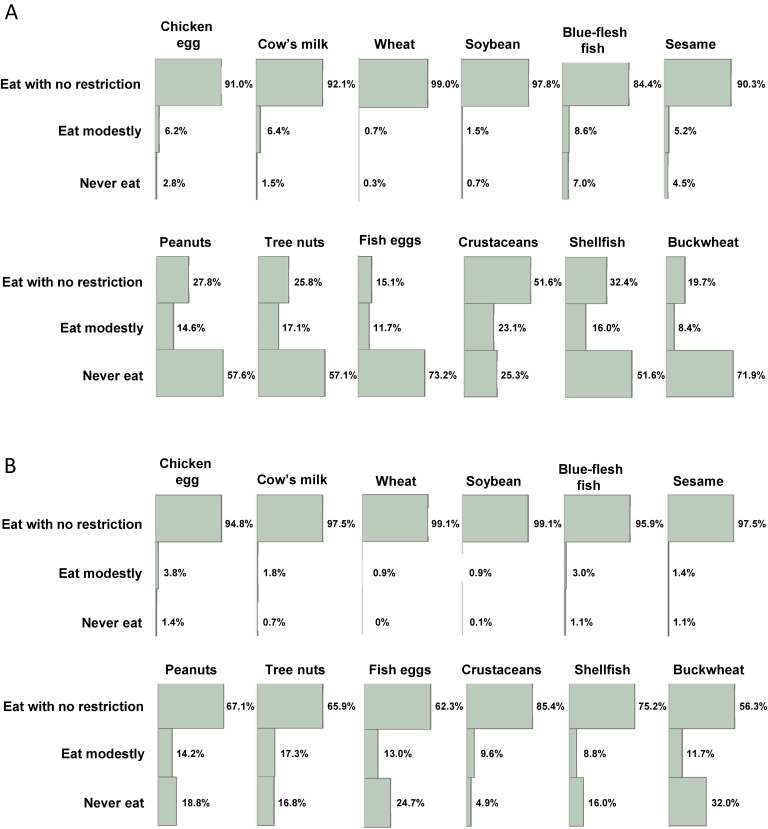
Rates of restriction on allergenic food intake at 1.5 (A) and 3 years (B) of age

At the age of 1.5 years, chicken egg, cow’s milk, and wheat—which have been reported to be the top 3 food allergens in young children in Japan [12]—were being completely avoided (“never eat”) in 2.8%, 1.5% and 0.3% of the children, respectively (Fig. 1A). On the other hand, peanuts, tree nuts, fish eggs, crustacean, shellfish, and buckwheat, which are much less common allergens compared to chicken egg and cow’s milk, were being completely avoided in 57.6%, 57.1%, 73.2%, 25.3%, 51.6%, 71.9%, respectively. Blue-flesh fish and sesame, which are rare allergens, were avoided in 7.0% and 4.5%, respectively. Other foods, including white-flesh fish, chicken, beef, pork, fruits and vegetables, were being avoided in less than 1% of the children (data not shown).

At age 3 years, the rate of avoidance decreased for all foods (Fig. 1B), but peanuts, tree nuts, fish eggs, shellfish, and buckwheat were still being avoided at high rates of 18.8%, 16.8%, 24.7%, 16.0%, and 32.0%, respectively.

### Factors associated with food avoidance

**Fig. 2:**
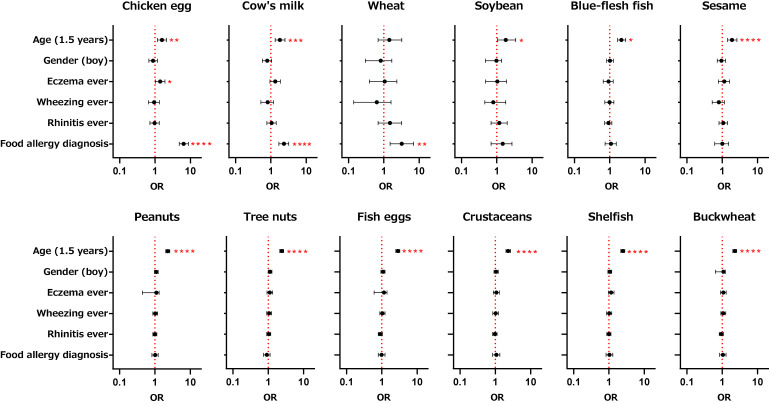
Associations of food avoidance with age, gender and allergic diseases by ordinal logistic regression. Separate ordinal logistic regression models were used to determine associations between ordinal outcomes of each food avoidance and possible risk factors of food allergy. Models were adjusted for covariates including age, gender, and questionnaire-identified allergic diseases. OR: odds ratios (horizontal bars indicate 95%CI). *P < 0.05, **P < 0.01, ***P < 0.001, ****P < 0.0001

Ordinal logistic regression analysis using levels of food avoidance as the objective variable and age, gender, and each allergic disease (eczema, wheezing, rhinitis, food allergy diagnosis) as explanatory variables showed that avoidance of each of chicken egg, cow’s milk, and wheat was associated with a food allergy diagnosis (Fig. 2). Chicken egg avoidance also showed a statistically significant association with “eczema ever”. Avoidance of other foods, including peanuts and tree nuts, was not associated with any allergic disease. However, age (1.5 years) was significantly associated with avoidance of chicken eggs, cow’s milk, soybean, blue-flesh fish, sesame, peanuts, tree nuts, fish eggs, crustaceans, shellfish, and buckwheat, indicating that avoidance of these foods was more common at 1.5 years (Fig. 2). Stratified analyses by age were also done. Avoidance of chicken egg and cow’s milk was associated with food allergy diagnosis in both 1.5 years and 3 years old groups with odds ratio (95%CI) being 6.2 (4.3–9.1), 7.3 (4.5–12.9), 1.9 (1.3–2.8), and 3.6 (2.0–6.5), respectively. Avoidance of cow’s milk was significantly associated with “eczema ever” in 1.5 years old group with odds ratio (95%CI) of 1.5 (1.01–2.19). Avoidance of other foods had no association with the clinical factors (data not shown).

### Replies to administrative questions

**Fig. 3:**
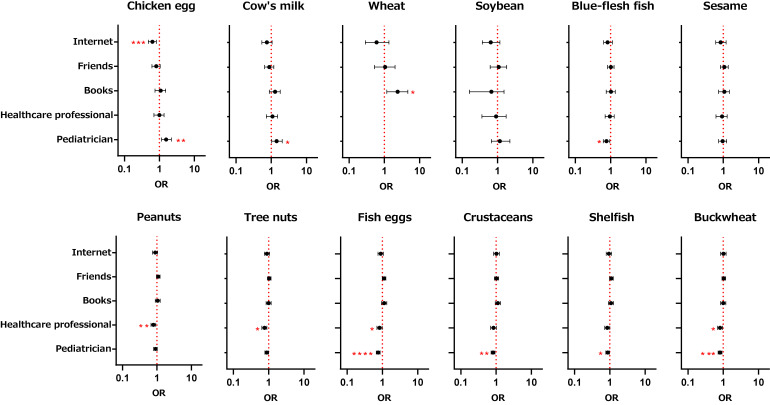
Association of food avoidance with the caregivers’ source(s) of medical information by ordinal logistic regression. Separate ordinal logistic regression models were used to determine associations between ordinal outcomes of each food avoidance and the sources of medical information that caregiver sought. Models were adjusted for covariates of medical information sources that caregivers chose in the questionnaire. OR: odds ratios (horizontal bars indicate 95%CI). *P < 0.05, **P < 0.01, ***P < 0.001, ****P < 0.0001 ORs (95%CI) for “Healthcare professional” and “Pediatrician” to wheat avoidance outcomes are not shown because the calculations results were unstable.

Table E3 summarizes the responses to administrative questions regarding the medical needs relating to allergy and COVID-19. Among them, the top 2 replies to “What do you do when you need to know something about allergies?” (= the caregivers’ sources of information regarding allergy) were “Search the Internet” at 84.2%, and “Consult a pediatrician” at 67.9%. Ordinal logistic models were constructed to investigate whether avoidance of foods had any association with the caregivers’ information source(s) (Fig. 3). Avoidances of peanuts, tree nuts, fish eggs, and buckwheat were significantly associated with a low probability of a healthcare professional as a source of information. Avoidances of fish eggs, crustaceans, shellfish, blue-flesh fish, and buckwheat were similarly significantly associated with a low probability of a pediatrician as the source. On the other hand, avoidance of chicken egg and cow’s milk was associated with high probability of a pediatrician as the source. Chicken egg avoidance had significantly low probability of the Internet as the source.

Stratified analyses by age showed that avoidance of chicken egg was associated with high probability of a pediatrician as the source with odds ratio (95%CI) of 1.7 (1.2–2.7) and that, conversely, avoidance of fish egg was associated with low probability of a pediatrician as the source with odds ratio (95%CI) of 0.72 (0.58–0.88). No other significant associations were found in the stratified analyses (data not shown).

Here, a health care professional means a nurse, pharmacist or others (including a physician) with whom a respondent is acquainted, while a pediatrician has a more formal meaning and indicates consultation in a professional setting.

## Discussion

In the present study, we identified the reality of young children’s dietary habits in avoiding certain allergenic foods in a general population in Japan. Caregivers avoided peanuts, tree nuts, fish eggs, shellfish, crustaceans, and buckwheat to a high percentages of the children. On the other hand, the avoidance rates for chicken egg, cow’s milk and wheat, which are the top 3 foods causing allergies in Japan [12], were much lower and comparable to the expected prevalence.

Avoidance of each of the latter 3 foods was positively associated with a food allergy diagnosis and eczema, indicating that the avoidance was due to the presence of actual food allergy. In particular, caregivers avoiding chicken egg and milk chose a pediatrician as a source of medical information, and for chicken egg avoidance, they tended not to choose the Internet, where a mixture of authenticity and falsehood is to be expected. These results suggests that these caregivers eliminated the foods on solid grounds.

On the contrary, the former foods including peanut and tree nuts showing high avoidance rates had no association with allergy risk factors, suggesting that the avoidance was not based on an existing food allergy. In addition, the caregivers who avoided giving those foods to their children had a low probability of seeking allergy information from healthcare professionals or pediatricians, suggesting that the avoidance may not be based on correct medical information. Although these results may simply reflect “normal” Japanese eating habits, we considered it important to document the current situation because it may be relevant to the future epidemiology of food allergy.

Recent epidemiological studies have greatly influenced the concept of the pathogenesis of food allergy, especially of peanut allergy. Namely, it was reported that the prevalence of peanut allergy was far lower among Israeli Jews, who are commonly fed peanuts from infancy, than among British Jews, who are not commonly fed peanuts in infancy [6]. Subsequent randomized intervention studies demonstrated that early consumption of peanuts significantly prevented peanut allergy development compared with “traditional” delayed introduction [8, 21]. Conversely, household consumption of peanuts was shown to be significantly higher in children with peanut allergy in the United Kingdom [22]. Also, peanut protein levels, with biological allergenicity, in house dust with which infants were in daily contact correlated positively with household peanut consumption in the United States, when peanuts not been given to young children for fear of peanut allergy [23]. Collectively, environmental exposure of infants to peanut protein/allergens [24], even when they are not being fed peanuts, may be a risk for developing peanut allergy.

Unlike in Western countries, peanuts were not traditionally widely eaten in Japan. However, the health benefits [25–27] of peanuts and tree nuts are now better appreciated, and peanut consumption among adults appears to be increasing. On the other hand, it was recently reported that the proportion of tree nuts in food-induced anaphylaxis has increased in Japan [28]. Our present study demonstrated that peanuts and tree nuts are avoided by the caregivers of many young children. If what has been observed in the United Kingdom and the United States were to occur in Japan, the current situation may point to a risk of peanut or tree nuts allergy development in Japan [29]. In the future, the prevalence of allergies to peanuts as well as tree nuts and other highly avoided foods should be carefully monitored.

From the nutritional aspect, a balanced diet is recommended for all ages in Japan [30]. The nutritional benefit of peanuts, tree nuts, fish, and shellfish, especially due to their high content of omega-3 fatty acid, has been widely reported [31–35]. Conversely, there are no known undesirable effects of these foods unless a child has confirmed allergies to them. Although it is not known whether the food avoidance found in this study caused any nutritional problems for the children, a nutritional survey is warranted.

As yet another aspect, peanuts and tree nuts are potential hazards as airway foreign bodies in children. The Japan Pediatric Society issued a public announcement that intact peanuts and other beans, etc., should not be given to children under 4 years of age to prevent fatal accidents due to airway foreign bodies [36]. It is possible that some caregivers did not give their children peanuts and tree nuts for fear of accidental aspiration. However, peanut butter and other non-granular forms of peanuts and tree nuts should be safe.

This study was conducted with an administrative objective of identifying medical needs regarding allergic diseases, and the results (Table E3) will be utilized to develop policies to combat allergic diseases. Since the survey was performed during the COVID-19 pandemic, the caregivers’ concerns about their children’s health and allergy related to the pandemic were also identified.

Below, we review recent epidemiological studies on pediatric allergic diseases in Japan. A nationwide survey of approximately 60,000 people (the Japan Environment and Children’s Study: JECS) reported atopic dermatitis in 18.5% of 1-year-olds and 14.7% of 3-year-olds, and physician-diagnosed food allergies in 6.6% and 6.3% of respondents, respectively [37]. In an online nationwide survey of 633 children aged 2–6 years, 16.7% of caregivers reported atopic dermatitis by ISAAC, and 16.7% also reported physician-diagnosed food allergy [38]. A survey of 236 caregivers in Ishikawa Prefecture reported physician-diagnosed atopic dermatitis in 6.7% of children at 1.5 years and in 12.1% at 3 years [39]. Regarding wheezing, the prevalence by age 3 was at 30.2% [40] in the JECS cohort, based on the ISAAC questionnaire. The prevalence of physician-diagnosed asthma by age 3 was reported to be 9.7% [40]. Regarding allergic rhinitis, a nationwide survey of otolaryngologist families with children ages 0–4 years reported 5.1% having perennial allergic rhinitis and 3.8% having cedar pollinosis [41]. Overall, the results of the present study involving 771 children at 1.5 years and 832 at 3 years were comparable to those of previous reports except for rhinitis, which may have been less specific than in the above otolaryngologist-based survey. We believe that this study adds important new epidemiological data to the above body of knowledge.

This study has a number of limitations. First, it was conducted in 2 local cities in Japan and may not represent the reality of Japan as a whole. However, the caregivers who joined the study represented over 90% of the participants in health checkups for young children, and the identified prevalences of allergic diseases were comparable to in the previous epidemiological studies in Japan. In addition, the identified allergic diseases showed significant associations with each other, in agreement with previous findings [42]. Therefore, the population included in the study may be representative of the general child population in Japan. Of note, delayed introduction of possible allergenic food to infants was also reported as interim results (unpublished) from JECS [43]. Second, we did not collect information about the reasons why the caregivers withheld a certain food; the questions about food allergy did not specify individual foods to which the child was allergic; and children were not tested for their sensitization status to foods. Lack of the above information may have led to incorrect conclusions. Third, nutritional evaluation was not performed. It would be important to know whether avoiding these potentially harmful allergenic foods leads to a harmful nutritional outcome. Fourth, in our multivariate logistic analyses, not all possible confounding factors were included because we used responses to the questionnaire that did not ask for personal information.

## Conclusions

Many kinds of foods, namely, peanuts, tree nuts, fish eggs, shellfish, crustaceans, and buckwheat, were avoided by caregivers for high percentages of young children at 1.5 and 3 years old in a general population in Japan. Since recent epidemiological and interventional studies suggest that delayed introduction of certain foods such as peanut may increase the risk of developing food allergy, the reality we identified here will have to be carefully analyzed in relation to food allergy development in the future.

## Abbreviations

COVID-19: Coronavirus disease 19
ISAAC: International Study of Asthma and Allergies in Childhood
JECS: the Japan Environment and Children’s Study

## Supplementary information

**Additional file 1:** **Table E1** The Survey Questionnaire. **Table E2** Association of each allergic disease with age, gender and comorbid allergies. **Table E3** Replies to questions about sources of information for allergies, requests to prefectural government, visiting a pediatrician during the pandemic, and concerns regarding allergies in relation to COVID-19.

## Declarations

### Availability of data

Data are available from the authors on request.
