# Supplementary material for: Intake of allergenic foods at 1.5 years and 3 years of age in a general child population in Japan: a cross-sectional study
Source: Environ Health Prev Med. 2023 Jan 21;28:6. doi: 10.1265/ehpm.22-00213 (PMC9884563; doi:10.1265/ehpm.22-00213)
Supplement: Supplementary file 1 — Additional file 1: Table E1 The Survey Questionnaire. Table E2 Association of each allergic disease with age, gender and comorbid allergies. Table E3 Replies to questions about sources of information for allergies, requests to prefectural government, visiting a pediatrician during the pandemic, and concerns regarding allergies in relation to COVID-19. [file ehpm-28-006-s001.docx]

**Table E1** The Survey Questionnaire

1. Age (1.5 and 3 years)
2. Gender
3. Modified ISAAC questionnaire
   1. Has your child ever had wheezing or whistling in the chest at any time in the past?
   2. Has your child ever had a problem with sneezing, or a runny or blocked nose when he/she DID NOT have a cold or the flu??
   3. In the past 12 months, has this nose problem been accompanied by itchy-watery eyes?
   4. Has your child ever had an itchy rash that came and went for at least 6 months?
   5. Has your child ever been diagnosed with a food allergy?
4. Questions regarding food intake
   Please fill in using the following marks: Ⅹ for foods that your child has never eaten because you worry about food allergies or he/she has been diagnosed with a food allergy; Δ for foods that you give him/her in modest amount for the above reasons; and ○ for foods that your child eats without any restriction.
    ( ) Chicken eggs, ( ) Cow’s milk, ( ) Wheat, ( ) Soy, ( ) Peanuts, ( ) Tree nuts
    ( ) Blue-flesh fish, ( ) White-flesh fish, ( ) Chicken, ( ) Beef, ( ) Pork
    ( ) Fruits, ( ) Vegetables, ( ) Sesame, ( ) Fish eggs such as salmon roe,
    ( ) Shrimps and crabs, ( ) Shellfish, ( ) Buckwheat, ( ) Others
5. Questions about allergic disease care
   1. What do you do when you need to know something about allergies? Please select all that apply.
      - Search the Internet
      - Ask a friend
      - Read a book
      - Ask a healthcare professional I know
      - Visit a pediatrician and ask
      - Other
   2. What do you expect the prefectural government to provide for your needs when you consult about allergies? Please select all that apply.
      - I would like to be provided with correct information on which medical facility to see.
      - I would like to be provided with easy access to consultation such as a telephone hotline before seeing a doctor.
      - I would like to be provided with a reliable internet site.
      - I would like to be informed of events where I can consult about allergies.
      - I would like to be provided with the names and addresses of of medical facilities that I can easily visit.
      - Others
6. Questions regarding the COVID-19 pandemic
   1. What are your thoughts on seeing a pediatrician during the COVID-19 pandemic? Please choose all that apply.
      - I don't want to visit a pediatrician because I am afraid that my child might catch infections even if he/she is not very sick.
      - I don't worry about visiting a pediatrician if children with and without infectious diseases are in different sections.
      - I don’t worry about visiting a pediatrician if the time-frame of examination is different between children with and without infectious diseases.
      - I visit a pediatrician whenever I am concerned about my child’s health.
      - Other
   2. What are your concerns regarding allergies in relation to COVID-19? Please select all that apply.
      - I am hesitant to visit a pediatrician for minor symptoms.
      - I am hesitant to visit a pediatrician when food allergy symptoms occur.
      - I fear that my child has gotten COVID-19 when he/she wheezes.
      - I fear that my child will become seriously ill with COVID-19 because he/she has allergies.
      - I fear that my child may have some abnormality in sense of smell or taste when he/she has persistent nasal symptoms.
      - Other

**Table E2** Association of each allergic disease with age, gender and comorbid allergies

| Outcome | Factor | OR (95%CI) | P-value |
| --- | --- | --- | --- |
| Food allergy diagnosis | Age (1.5 years old) | 0.92 (0.65-1.32) | NS |
|  | Gender (boy) | 1.06 (0.74-1.50) | NS |
|  | Eczema ever | 3.88 (2.64-5.71) | <.0001 |
|  | Wheezing ever | 1.65 (1.10-2.47) | 0.02 |
|  | Rhinitis ever | 0.87 (0.59-1.27) | NS |
|  | Current rhinoconjunctivitis | 0.87 (0.43-1.78) | NS |
| Wheezing ever | Age (1.5 years old) | 0.63 (0.48-0.84) | <.0001 |
|  | Gender (boy) | 1.30 (0.99-1.72) | NS |
|  | Eczema ever | 1.88 (1.33-2.66) | <.0001 |
|  | Food allergy diagnosis | 1.63 (1.08-2.44) | 0.02 |
|  | Rhinitis ever | 2.93 (2.20-3.91) | <.0001 |
|  | Current rhinoconjunctivitis | 1.04 (0.64-1.70) | NS |
| Eczema ever | Age (1.5 years old) | 1.34 (0.98-1.83) | NS |
|  | Gender (boy) | 1.15 (0.84-1.57) | NS |
|  | Wheezing ever | 1.88 (1.33-2.66) | <.0001 |
|  | Food allergy diagnosis | 3.87 (2.63-5.70) | <.0001 |
|  | Rhinitis ever | 1.90 (1.37-2.64) | <.0001 |
|  | Current rhinoconjunctivitis | 1.43 (0.83-2.45) | NS |
| Rhinitis ever | Age (1.5 years old) | 0.88 (0.70-1.10) | NS |
|  | Gender (boy) | 1.08 (0.86-1.35) | NS |
|  | Eczema ever | 2.08 (1.52-2.85) | <.0001 |
|  | Food allergy diagnosis | 0.82 (0.57-1.18) | NS |
|  | Wheezing ever | 2.86 (2.18-3.76) | <.0001 |

OR: odds ratio; NS: not significant

Separate binary logistic regression models were used to determine associations between questionnaire-identified allergic diseases and known risk factors. Models were adjusted for covariates that are known to be associated with childhood allergic diseases, including age, gender, and other allergic diseases identified with questionnaire.

**Table E3** Replies to questions about sources of information for allergies, requests to prefectural government, visiting a pediatrician during the pandemic, and concerns regarding allergies in relation to COVID-19

| What do you do when you need to know something about allergies? | N (%) |
| --- | --- |
| Search the Internet | 1276 (84.2%) |
| Ask a friend | 532 (34.0%) |
| Check it out in a book | 191 (12.4%) |
| Ask a healthcare professional I know | 219 (14.2%) |
| Consult a pediatrician | 1047 (67.9%) |
| Other | 22 (1.4%) |
| What do you expect the prefectural government to provide for your needs when you consult about allergies? | N (%) |
| Information on which medical facility to see | 732 (48.3%) |
| Easily accessible consultation before seeing a doctor | 710 (46.9%) |
| Reliable Internet sites | 768 (50.7%) |
| Events for allergy consultation | 133 (8.8%) |
| Easy-to-visit medical facilities | 727 (48.0%) |
| Other | 13 (0.8%) |
| What are your thoughts on visiting a pediatrician during the COVID-19 pandemic? | N (%) |
| I don't want to visit a pediatrician because I am afraid that my child might catch an infection even if he/she is not very sick. | 885 (57.8%) |
| I don't worry about visiting a pediatrician if children with and without infectious diseases are in different sections. | 820 (53.6%) |
| I don’t worry about visiting a pediatrician if the time-frame of examination is different between children with and without infectious diseases. | 476 (31.0%) |
| I visit a pediatrician whenever I am concerned about my child’s health. | 442 (28.9%) |
| Other | 17 (1.1%) |
| What are your concerns regarding allergies in relation to COVID-19? | N (%) |
| I am hesitant to visit a pediatrician for minor symptoms. | 862 (65.0%) |
| I am hesitant to visit a pediatrician when food allergy symptoms occur. | 89 (6.7%) |
| I fear that my child has gotten COVID-19 when he/she wheezes. | 370 (27.9%) |
| I fear that my child will become seriously ill with COVID-19 because he/she has allergies. | 327 (24.6%) |
| I fear that my child may have some abnormality in sense of smell or taste when he/she has persistent nasal symptoms. | 344 (25.9%) |
| Other | 19 (1.4%) |
